# Corrosion Behavior and Discharge Performance of Germanium and Lanthanum Co-Doped AZ61 Alloy Anodes for Mg–Air Batteries

**DOI:** 10.3390/ma19071305

**Published:** 2026-03-25

**Authors:** Qi Liu, Baosheng Liu, Yuezhong Zhang, Shaohua Zhang, Pengpeng Wu

**Affiliations:** School of Materials Science and Engineering, Taiyuan University of Science and Technology, Taiyuan 030024, China

**Keywords:** AZ61, Ge element, Mg–air battery, corrosion behavior, discharge performance

## Abstract

**Highlights:**

- 0.7Ge alloy exhibits superior corrosion resistance and discharge performance for Mg–air batteries.
- Grain refinement and uniform second phase promote a loose, detachable discharge product layer.
- An optimal Ge content balances micro-galvanic corrosion to enable uniform dissolution and inhibit the chunk effect.

**Abstract:**

Magnesium–air battery anodes suffer from self-corrosion, chunk effect, and poor removal of discharge products, resulting in low anode efficiency. Although various modification strategies for Mg anodes have been reported, the effects of Ge content on the microstructure and performance of AZ61 Mg anodes at a fixed La content remain unclear. In this study, AZ61-1La-*x*Ge alloys (*x* = 0, 0.25, 0.7, and 0.9 wt.%) were prepared, and their microstructure, corrosion behavior, and discharge performance after solution treatment were systematically investigated. Among them, AZ61-1La-0.7Ge exhibited the best overall performance, mainly due to the appropriate addition of Ge, which promoted a uniform distribution of secondary phases and grain refinement, thereby suppressing self-corrosion and chunk effect, improving discharge uniformity, and enhancing anode utilization by facilitating the formation of a loose discharge product layer. This study provides a basis for optimizing the Ge content in La-modified AZ61 Mg alloy anodes.

## 1. Introduction

The excessive reliance on non-renewable energy sources such as coal, gasoline, etc., in conventional energy structures has significantly contributed to greenhouse gas emissions and associated environmental and health hazards [1,2,3,4]. Therefore, the shift toward more sustainable and eco-conscious energy systems has become imperative [5,6,7,8]. Against this backdrop, research into electrochemical energy storage systems has been accelerated considerably [9,10,11,12]. Metal–air batteries have attracted extensive attention as electrochemical energy-storage systems, in which metal functions as the negative electrode and oxygen supplied from ambient air acts as the positive-electrode reactant [13]. Their numerous benefits encompass elevated energy density, straightforward configuration, plentiful raw materials, and environmental friendliness, positioning them as a promising option for future energy applications [14,15,16,17]. Common examples of metal–air batteries are Li–air batteries, Zn–air batteries, and Mg–air batteries [18].

Mg–air batteries (MABs), in comparison with other types of metal–air batteries, exhibit multiple advantages such as rich magnesium reserves, low production cost, light weight, environmental compatibility, high safety level, as well as a high theoretical specific energy and a theoretical discharge voltage [19,20,21]. These characteristics make MABs promising candidates for powering devices in military, marine, and medical applications [22,23,24,25]. Magnesium anodes often exhibit discharge capabilities that fall short of their theoretical values. This is partly due to their tendency to undergo self-corrosion in aqueous electrolyte environments [26]. During the discharge stage, the negative difference effect (NDE) contributes to the problem by significantly enhancing hydrogen evolution, thereby worsening anode degradation [27]. Moreover, non-uniform dissolution of the anode can cause partially unreacted material to separate from the electrode, a phenomenon commonly referred to as the chunk effect, thereby lowering anode utilization efficiency. Meanwhile, the corrosion and discharge products accumulate on the anode, impeding contact between the electrolyte and the anode matrix, thereby diminishing discharge activity and resulting in a decline in performance.

Therefore, enhancement of magnesium alloy anode behavior mainly relies on inhibiting self-corrosion, mitigating material loss associated with the chunk effect, and reducing the deposition of discharge products at the electrode interface. Alloying has been demonstrated to be an efficient route for mitigating these issues, and rare-earth elements, in particular, play a pivotal role in improving the discharge performance of magnesium-based anodes [28,29]. As an abundant and economical RE element, La is regarded as a promising candidate. Earlier studies found that the introduction of 5 wt.% La results in the development of a continuous network-like LaMg_12_ phase, which facilitates more uniform consumption of the magnesium substrate and accelerates the removal of reaction products from the surface, thereby enhancing specific capacity as well as anodic efficiency [30]. Furthermore, Liu et al. reported that the synergistic addition of Ca, Sm, and La refines the grains and the Mg_17_Al_12_ phase in AZ91 alloy, while promoting a more homogeneous distribution of secondary phases, resulting in enhanced discharge voltage stability and overall discharge performance [31]. According to Chen et al., adding La and Gd to the AZ80 alloy promoted the formation of fine Al-RE intermetallic phases within the matrix. These phases altered the morphology of Mg_17_Al_12_ and contributed to enhanced corrosion resistance together with improved discharge-product desorption [32].

As a group-IV element, Ge has been shown to effectively optimize the microstructure and electrochemical behavior of magnesium anodes by improving corrosion resistance, mitigating the negative difference effect and the chunk effect, and increasing discharge activity and energy density. For example, Chen et al. reported that ultra-high-purity Mg-Ge alloys exhibited a remarkably low corrosion rate of 0.15 mm·y^−1^ in 3.5 wt.% NaCl solution, with an anodic efficiency of 73% and an energy density of 2178 Wh·kg^−1^, demonstrating excellent performance [33]. Jiang et al. reported that the Mg-0.5Zn-0.2Ge alloy exhibited a more negative open-circuit potential, accompanied by clear suppression of both self-corrosion and the chunk effect. When this alloy was used as the anode in a Mg–air battery, the cell delivered higher discharge voltage and specific energy, highlighting the beneficial role of Ge as a microalloying element in primary Mg–air battery anodes [34]. It is worth noting, however, that the solubility of Ge in magnesium remains very limited. An excessive Ge content tends to promote the formation of abundant secondary phases, thereby strengthening galvanic corrosion of the Mg matrix and exacerbating the chunk effect, with a consequent decline in discharge behavior. Therefore, precise control of the Ge content is crucial to achieving an optimal balance between microstructural regulation and electrochemical performance [35].

AZ-series magnesium alloys are commercially available Mg alloys with reliable performance and relatively low cost, and they show great potential as anode materials for magnesium–air batteries. Previous studies have shown that appropriate alloying modification can refine the grain structure and promote a fine and homogeneous distribution of secondary phases, thereby improving the corrosion resistance and discharge performance of Mg anodes [36]. Among various alloying elements, La and Ge have been reported to exert beneficial effects on the microstructure and electrochemical behavior of Mg alloys. However, under a fixed La content, the effects of Ge content on the microstructural evolution, corrosion behavior, and discharge performance of AZ61 Mg alloy anodes have not yet been systematically clarified. Therefore, in this study, commercial AZ61 alloy was selected as the base alloy, and AZ61-1La-*x*Ge alloys with different Ge contents were prepared and solution-treated to elucidate the effect of Ge content on the anode performance of AZ61-1La alloys, and to further clarify the roles of secondary-phase distribution and grain size in anode activation and discharge behavior.

## 2. Materials and Methods

### 2.1. Materials Preparation

The AZ61-1La-*x*Ge alloys (*x* = 0, 0.25, 0.7, and 0.9 wt.%) prepared in this study were synthesized via conventional gravity casting. The starting materials comprised AZ61 alloy ingots, Mg-30 wt.% La master alloy, and high-purity Ge particles with 99.99 wt.% purity. The alloy was prepared by melting in a resistance crucible furnace with CO_2_ + SF_6_ as the protective gas, and the melting temperature was maintained at 750 °C. The AZ61 alloy was completely melted initially, followed by the gradual addition of accurately weighed Mg-30La master alloy and Ge particles, with continuous stirring applied to achieve uniform composition. After degassing and slag skimming, the melt was transferred into a steel mold maintained at 250 °C, followed by air cooling to obtain cast bars with a diameter of 100 mm. The cast bars were sectioned into several cubic specimens using a wire electrical discharge machining (Wire EDM) system. The samples underwent solution treatment at 420 °C for 24 h, followed by rapid water quenching to promote a more homogeneous distribution of alloying elements in the matrix and achieve full homogenization. The actual chemical compositions of the alloys were analyzed using inductively coupled plasma optical emission spectrometry (ICP-OES, PE-200, Shelton, CT, USA), and each alloy was measured three times independently to ensure reproducibility, with the values listed in Table 1 reported as the averages of these measurements.

### 2.2. Microstructure Characterization

Before microstructural observation, the samples were cleaned ultrasonically in ethanol, ground to 2000 grit, polished, and then etched in an ethanol solution containing 4 vol.% nitric acid. Microstructural observation was performed by field-emission scanning electron microscopy (FE-SEM; ZEISS Sigma 300, Oberkochen, Germany), whereas phase identification and compositional analysis were carried out using energy-dispersive spectroscopy (EDS; Hitachi Regulus 8230, Tokyo, Japan), X-ray diffraction (XRD; Rigaku SmartLab SE, Tokyo, Japan), and transmission electron microscopy (TEM; FEI Tecnai G2 F30, Hillsboro, OR, USA). Furthermore, the potential difference between the matrix and secondary phases was characterized by SKPFM (Dimension Icon, Bruker, Billerica, MA, USA).

### 2.3. Hydrogen Evolution Immersion

**Figure 1:**
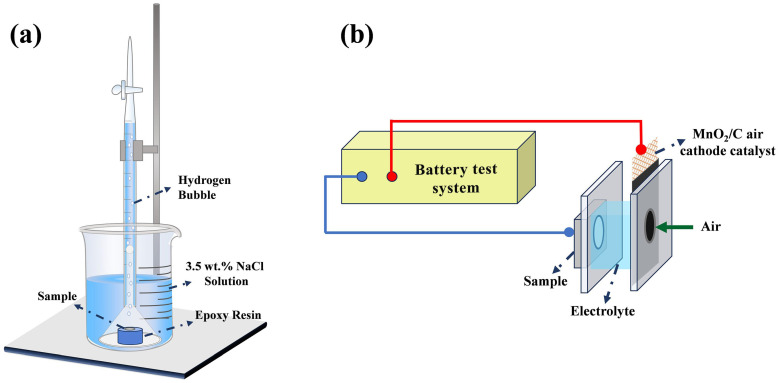
(**a**) Schematic illustration of the hydrogen evolution setup, and (**b**) schematic illustration of the discharge setup.

For the hydrogen evolution test, the samples were mounted in epoxy resin, leaving only a 1 cm^2^ area exposed to the solution. The exposed surface was then ground with silicon carbide papers until it appeared bright, after which it was cleaned, dried, and weighed. The prepared samples were immersed in 3.5 wt.% NaCl solution at 25 °C for 120 h. As illustrated in Figure 1a, a burette and an inverted funnel were positioned above the specimen to capture the hydrogen evolved during the immersion test. Following the experiment, corrosion products on the sample surface were cleaned off using a 200 g/L chromic acid solution. The corrosion rate was determined according to Equations (1) and (2) [37,38]. To ensure data reliability, each test condition was repeated with at least three parallel samples.

(1) $$P_{H}=2.279V_{H}$$

(2) $$P_{W}=2.10\frac{W_{a}-W_{b}}{At}$$

where *P_H_* is the hydrogen evolution corrosion rate, mm y^−1^. *V_H_* denotes the volume of hydrogen evolved, mL cm^−2^ day^−1^. *P_W_* is the mass loss corrosion rate, mm y^−1^. *W_a_* and *W_b_* are the sample masses before and after corrosion product removal, respectively, mg. *A* is the exposed surface area of the sample, cm^2^. And *t* is the immersion time, d.

### 2.4. Electrochemical Test

A conventional three-electrode configuration was employed for the electrochemical tests. The AZ61-1La-*x*Ge alloy specimens served as the working electrodes, with the exposed area fixed at 1 cm^2^; a platinum sheet and a saturated calomel electrode (SCE) were used as the counter and reference electrodes, respectively. 3.5 wt.% NaCl solution was used for all tests. Two electrochemical methods were employed for characterization, namely potentiodynamic polarization (PDP) and electrochemical impedance spectroscopy (EIS). To ensure that the sample test surface was stable, a 30 min open circuit potential (OCP) test was performed, and EIS were obtained at a frequency range of 0.1 Hz to 100 kHz with a voltage amplitude of 10 mV relative to the OCP. PDP analysis was performed within a voltage window ranging from −2 V to −1 V, using a scanning rate of 1 mV/s. To improve the reliability of the data, all tests were repeated on at least three parallel specimens. The corrosion rate was determined using Equation (3) [39]:

(3) $$P_{i}\;=\;22.85i_{corr}$$

where *P_i_* refers to the corrosion rate, mm year^−1^. While *i_corr_* denotes the corrosion current density, mA cm^−2^.

### 2.5. Discharge Testing

The discharge experimental setup was adapted from a conventional zinc–air battery configuration, employing the AZ61-1La-*x*Ge alloy as the anode, MnO_2_/C as the air cathode catalyst, and 3.5 wt.% NaCl solution functioned as the electrolyte. Discharge performance was assessed with a LAND battery testing system under constant current conditions. The working electrode had an exposed circular surface with a diameter of 1 cm, serving as the active area during discharge measurements. The samples were discharged at two different current densities (2 mA cm^−2^, 10 mA cm^−2^) for 10 h. The electrolyte was circulated during the discharge tests using a water pump with a nominal flow rate of 200 L h^−1^ to maintain a stable electrolyte concentration throughout the experiment. Prior to discharge, the working surface of the Mg alloy anode was immersed in the electrolyte for 5–10 min to stabilize the surface state and ensure stable performance during the discharge test. The diagram of the discharge setup is shown in Figure 1b. Discharge products were removed using a chromic acid solution at 200 g/L. Based on the corresponding mass loss, the anode efficiency, capacity density, and energy density of each alloy were calculated using Equations (4)–(6) [26,40]:

(4) $$\mathrm{Anodic}\;\mathrm{efficiency}\;\left(\%\right)\;=\;\frac{(i\;\times \;A\;\times \;t\;)\;\times \;M_{a}}{2F(W_{i}\;-W_{f})}$$

(5) $$\mathrm{Specific}\;\mathrm{capacity}\;\mathrm{density}\;(\mathrm{mAh}\;g^{-1})=\frac{i\;\times \;A\;\times \;t}{W_{i}-W_{f}}$$

(6) $$\mathrm{Specific}\;\mathrm{energy}\;\mathrm{density}\;(\mathrm{mWh}\;g^{-1})=\frac{\int_{0}^{t}U\;\times \;i\;\times \;dt}{W_{i}-W_{f}}$$

where *i* refers to the applied discharge current density, mA cm^−2^. *A* represents the anodic surface area involved in the discharge process, cm^2^. And *t* is the duration of discharge under a constant current, h. *Mₐ* stands for the atomic mass of the material, g mol^−1^. F is the Faraday constant, 96,485 C mol^−1^. The parameters *W_i_* and *W_f_* indicate the sample’s weight before and after the removal of corrosion products, respectively, g. *U* denotes the discharge voltage, V.

## 3. Results

### 3.1. Microstructures

**Figure 2:**
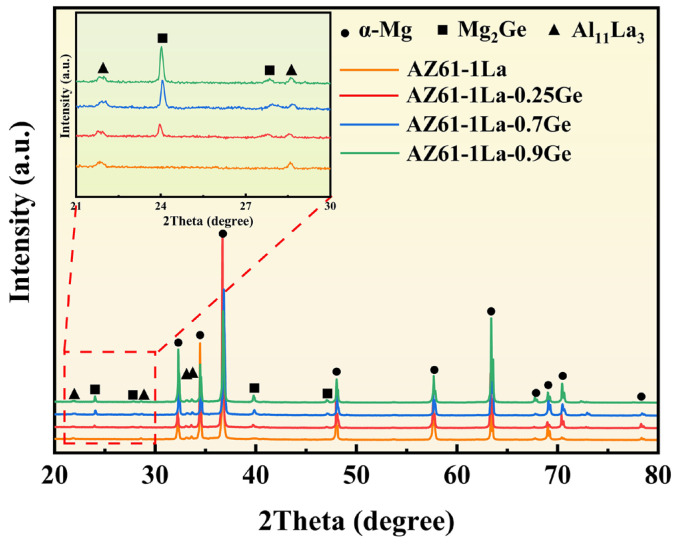
XRD patterns of AZ61-1La-*x*Ge alloy (*x* = 0, 0.25, 0.7, 0.9 wt.%).

**Figure 3:**
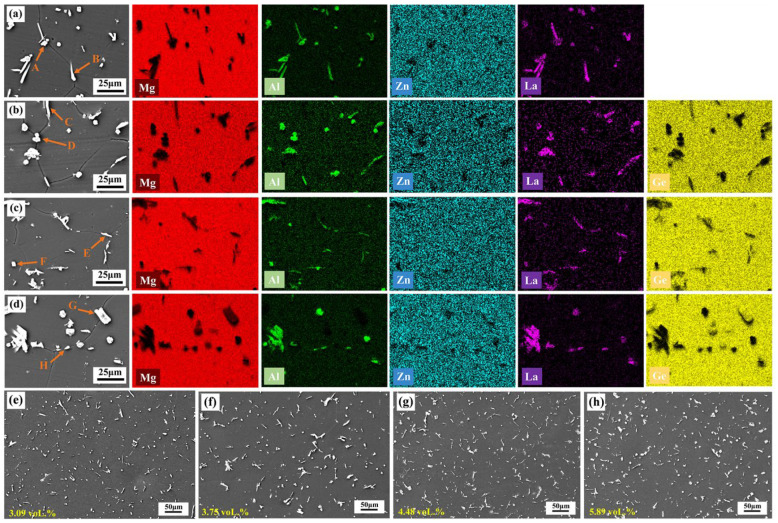
(**a**–**d**) EDS mappings of AZ61-1La-*x*Ge alloy: (**a**) 0 wt.%, (**b**) 0.25 wt.%, (**c**) 0.7 wt.%, and (**d**) 0.9 wt.%; (**e**–**h**) the SEM micrographs of AZ61-1La-xGe alloy: (**e**) 0 wt.%, (**f**) 0.25 wt.%, (**g**) 0.7 wt.%, and (**h**) 0.9 wt.%.

**Figure 4:**
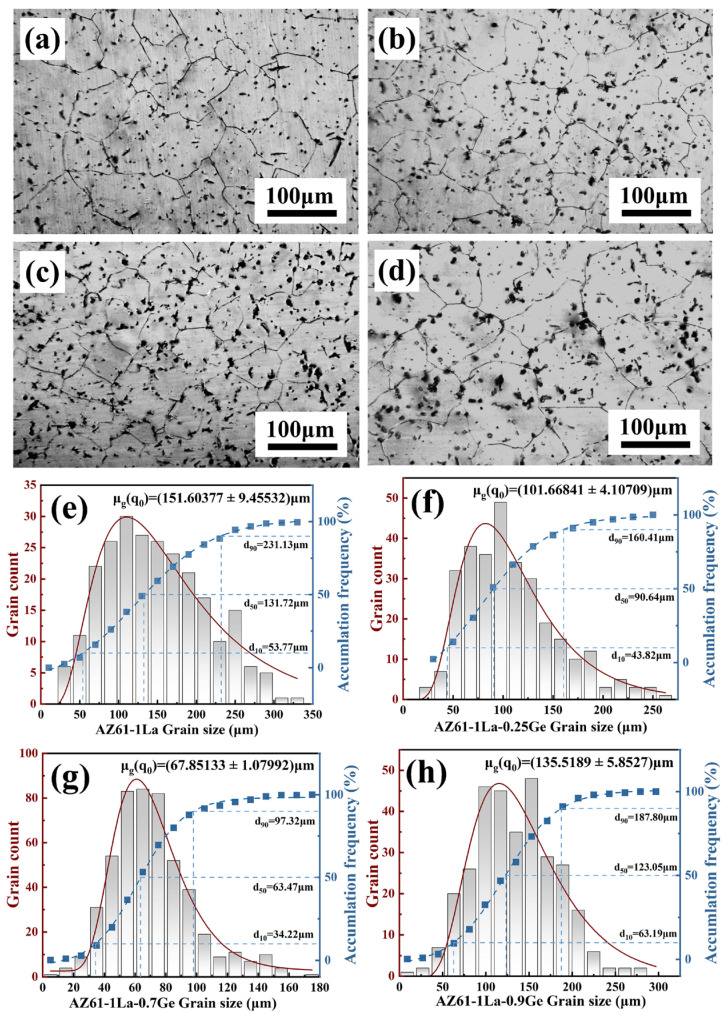
Optical micrographs and grain size distributions of AZ61-1La-*x*Ge alloys: (**a**) 0 wt.%, (**b**) 0.25 wt.%, (**c**) 0.7 wt.%, and (**d**) 0.9 wt.%, (**e**–**h**) statistical grain size distributions of the AZ61-1La-*x*Ge alloys.

Figure 2 presents the XRD patterns of AZ61-1La-*x*Ge (*x* = 0, 0.25, 0.7, and 0.9 wt.%) alloys. As can be seen from the figure, in addition to the α-Mg matrix, the AZ61-1La alloy also contains the Al_11_La_3_ phase. With the addition of Ge, the Mg_2_Ge phase is formed in the matrix, and as the Ge content increases, the intensity of the Mg_2_Ge diffraction peak gradually increases. The SEM morphologies and EDS elemental mapping results of the AZ61-1La-*x*Ge alloys are shown in Figure 3. The compositional data derived from spot analyses at the indicated locations are provided in Table 2. Combined with the XRD and EDS results, two main second-phase compounds, Al_11_La_3_ and Mg_2_Ge, are identified in the alloys. The solution-treated alloy exhibits a homogeneous distribution of secondary phases within the matrix. The overall volume fraction of secondary phases increases with increasing Ge content, indicating a progressive rise in the fraction of the Mg_2_Ge phase within the matrix. As observed in the optical micrographs in Figure 4, the average grain size of the four alloys decreases first and then increases with increasing Ge content. An appropriate Ge addition promotes the formation of fine, dispersed secondary phases that pin grain boundaries, thereby refining the grains. However, excessive Ge (0.9 wt.%) causes coarsening of the secondary phases, which weakens the grain-boundary pinning effect and leads to a rebound in grain size.

**Figure 5:**
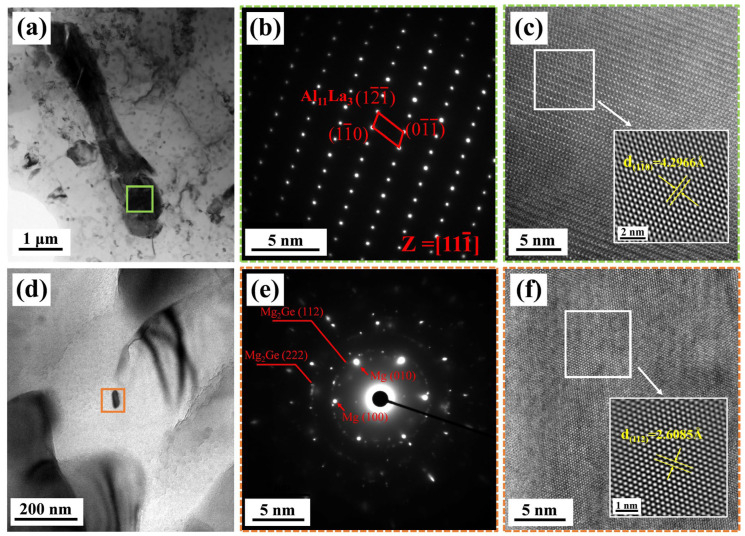
TEM analysis of the AZ61-1La-0.7Ge alloy, (**a**) Bright-field TEM image of Al_11_La_3_, (**b**) SAED pattern from the region marked in (**a**), (**c**) HRTEM images of Al_11_La_3_; (**d**) bright-field TEM image of Mg_2_Ge, (**e**) SAED pattern from the region marked in (**d**), (**f**) HRTEM images of Mg_2_Ge.

In order to further study the crystal structure of the second phase in the matrix, TEM analysis was conducted on the alloy. Given that the phase composition of alloys with different Ge contents is basically the same, the AZ61-1La-0.7Ge alloy with moderate Ge content was selected as a representative sample for characterization. As shown in Figure 5, HRTEM and SAED characterizations were conducted on the marked areas in Figure 5a,d. A SAED image of the bar phase in Figure 1a is displayed in Figure 5b. The rod-like phase observed in Figure 5a was identified as the Al_11_La_3_ phase based on the analysis of its corresponding Miller indices. A HRTEM image of the Al_11_La_3_ phase is displayed in Figure 5c, where the crystallographic spacing of 4.2966 Å corresponds to the $\left(1\bar{1}0\right)$ crystallographic plane of the Al_11_La_3_ phase, further illustrating that the bar phase in Figure 5a is the Al_11_La_3_ phase. Analysis of the SAED pattern of the nanoparticle in Figure 5d, as shown in Figure 5e, revealed distinct diffraction spots corresponding to the Mg matrix, along with diffraction rings attributed to the Mg_2_Ge (112) and Mg2Ge (222) planes. This confirms the presence of the nanoscale Mg_2_Ge phase. The HRTEM image in Figure 5f displays the lattice fringes of the Mg_2_Ge phase, with an interplanar spacing of 2.6085 Å, further substantiating the existence of the nanoscale Mg_2_Ge phase.

### 3.2. Corrosion Performance

**Figure 6:**
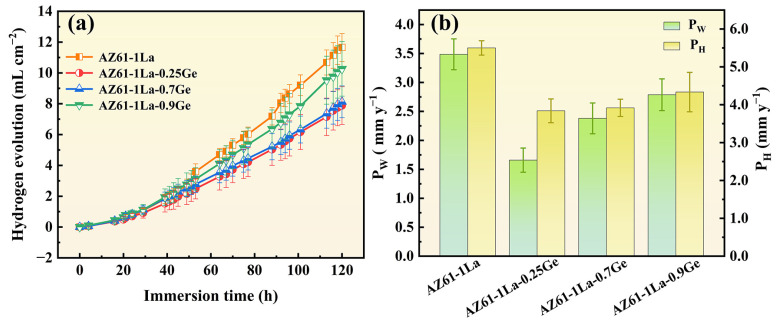
(**a**) Hydrogen evolution test of AZ61-1La-*x*Ge alloy immersed in 3.5 wt.% NaCl solution for 120 h. (**b**) Corrosion rates of AZ61-1La-*x*Ge alloys.

The relationship between hydrogen evolution volume and immersion time for AZ61-1La-*x*Ge alloys immersed in 3.5 wt.% NaCl at 25 °C is depicted in Figure 6a. In the first 30 h, all alloys exhibited low and stable corrosion rates due to the initial protective oxide layer. After 30 h, this layer degraded, leading to accelerated corrosion at varying degrees [41]. Hydrogen evolution increased linearly over 120 h, indicating a relatively steady corrosion process. Among the alloys, AZ61-1La-0.25Ge showed the lowest hydrogen evolution (7.88 mL cm^−2^), followed by 0.7Ge (8.12 mL cm^−2^). This indicates that trace Ge addition can effectively enhance the corrosion resistance. Corrosion rates measured after 120 h of continuous immersion at 3.5 wt.% NaCl solution is presented in Figure 6b, calculated by both hydrogen evolution (*P_H_*) and mass loss (*P_W_*) methods, showing consistent trends. Combining Figure 6a,b, the corrosion resistance of the alloys follows the order: AZ61-1La-0.25Ge > AZ61-1La-0.7Ge > AZ61-1La-0.9Ge > AZ61-1La.

**Figure 7:**
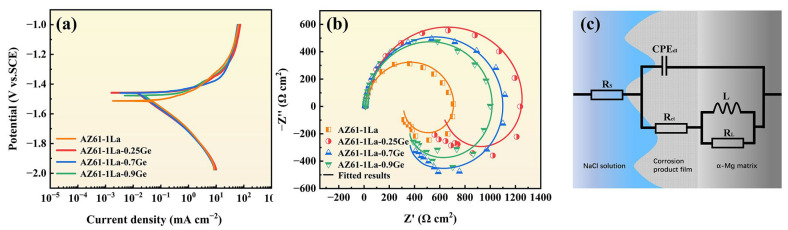
(**a**) Polarization curves of AZ61-1La-*x*Ge alloys in 3.5 wt.% NaCl solution, (**b**) Nyquist diagram of AZ61-1La-*x*Ge alloys, and (**c**) fitting circuit diagram of AZ61-1La-*x*Ge alloys.

Figure 7a shows PDP curves of AZ61-1La-*x*Ge alloys (*x* = 0, 0.25, 0.7, 0.9 wt.%) in 3.5 wt.% NaCl solution, and Table 3 summarizes the corresponding corrosion potentials (E_corr_) and corrosion current densities (I_corr_). In general, the lower I_corr_ and the more positive E_corr_ indicate better corrosion resistance. It can be observed from Table 2 that the AZ61-1La-0.25Ge alloy exhibits the lowest I_corr_ and the most positive E_corr_, indicating superior corrosion resistance, followed by the 0.7Ge alloy.

Figure 7b shows the Nyquist plots of AZ61-1La-*x*Ge alloys in 3.5 wt.% NaCl solution, while Figure 7c presents the corresponding equivalent circuit, and the fitted parameters are summarized in Table 4. All samples exhibit a high-frequency capacitive loop and a low-frequency inductive loop, indicating similar corrosion characteristics [42]. The high-frequency capacitive loop is mainly associated with the charge-transfer process and the non-ideal capacitive response of the surface corrosion product film/double layer, whereas the low-frequency inductive loop is generally related to the relaxation of adsorbed intermediates and the instability of the surface film, suggesting that the corrosion behavior of this system is governed by multiple interfacial processes. Combined with Figure 7b and Table 4, it can be seen that the AZ61-1La-0.25Ge alloy exhibits the largest capacitive arc radius as well as the highest R_ct_ and R_total_ values, indicating the strongest resistance to interfacial charge transfer and thus the best corrosion resistance [43,44]. In contrast, the AZ61-1La-0.7Ge alloy shows a slightly lower R_ct_, but higher L and R_L_ values, indicating a more pronounced low-frequency relaxation process and a surface state that is more readily maintained in an active condition during discharge [45]. It should be noted that, for Mg alloy systems showing an obvious low-frequency inductive loop, corrosion resistance cannot be evaluated solely on the basis of a single R_ct_ value, but should be assessed comprehensively by combining the Nyquist plot features and fitted parameters [46,47]. Therefore, the EIS results indicate that the AZ61-1La-0.25Ge alloy possesses the best corrosion resistance, whereas the AZ61-1La-0.7Ge alloy exhibits better discharge activity and overall discharge performance while maintaining relatively high corrosion resistance.

### 3.3. Discharge Performance

**Figure 8:**
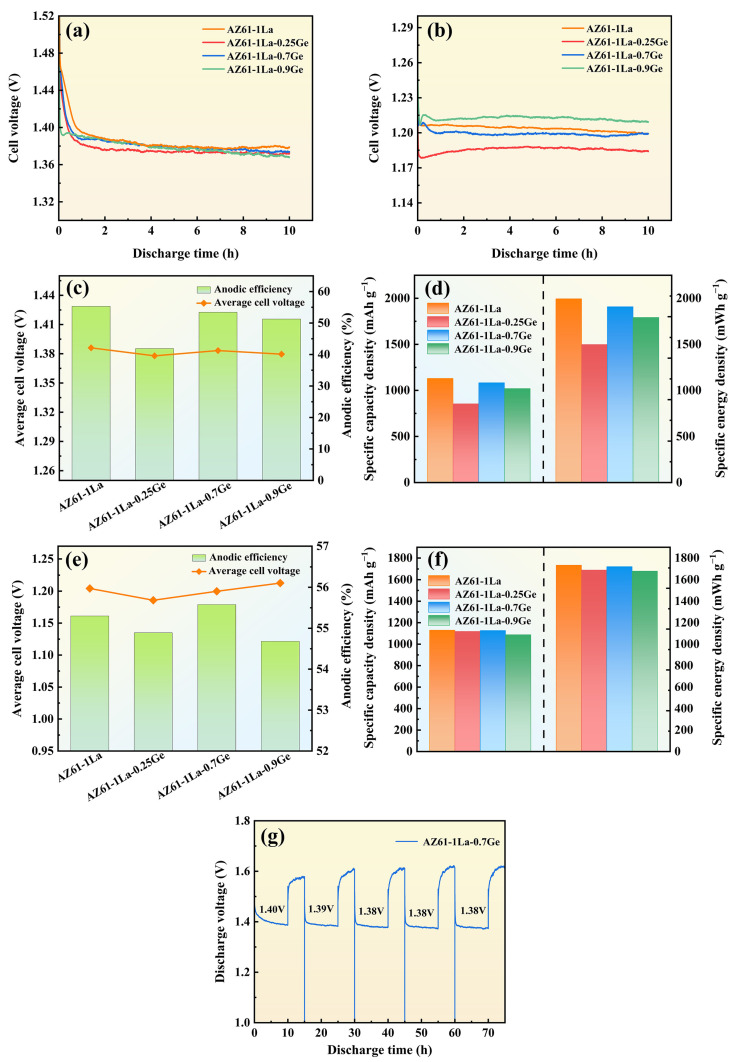
Discharge performance of AZ61-1La-*x*Ge (*x* = 0, 0.25, 0.7, 0.9 wt.%) alloys, (**a**) discharge curves after discharging at 2 mA cm^−2^ for 10 h, (**b**) discharge curves after discharging at 10 mA cm^−2^ for 10 h, (**c**,**d**) show the discharge performance of AZ61-1La-*x*Ge alloys at 2 mA cm^−2^, while (**e**,**f**) present the discharge performance of AZ61-1La-*x*Ge alloys at 10 mA cm^−2^, in subfigures (**d**,**f**), the specific capacity and specific energy are separated by dashed lines in the figure, (**g**) The intermittent discharge curve of AZ61-1La-0.7Ge alloy.

The 10 h continuous-discharge behavior of AZ61-1La-*x*Ge alloys at 2 and 10 mA cm^−2^ is illustrated in Figure 8, with the related discharge profiles shown in Figure 8a and Figure 8d, respectively. It can be inferred from the analysis that discharge tests at a current density of 2 mA cm^−2^ revealed that AZ61-1La-*x*Ge alloy stabilized after approximately 1 h, which implies the occurrence of a dynamic steady state between the formation and detachment of discharge products. This equilibrium enabled a stable voltage output during the subsequent discharge process [48].

Under the higher current density (10 mA cm^−2^), the discharge voltages of all alloys were generally lower than those at the lower current density, mainly due to the increased generation rate of discharge products under higher current loads. The swift build-up of these products resulted in a noticeable decline in discharge voltage [49]. Despite the overall decrease in voltage, the discharge profiles of the AZ61-1La-*x*Ge alloys under high current density demonstrated minimal fluctuations, suggesting a high degree of voltage stability. This behavior can be attributed to the intensified discharge reaction of the AZ61-1La-*x*Ge anodes under high current conditions, where the primary discharge reaction proceeds at a faster rate than the parasitic corrosion reactions. As a result, the effective response of the anode surface is enhanced, leading to more stable discharge voltages. Additionally, AZ61-1La-0.7Ge and AZ61-1La-0.9Ge alloys showed a brief voltage rise at the initial discharge period, which is then succeeded by a gradual decrease. This phenomenon is associated with the initially uncovered anode surface and a large number of active discharge sites. In the initial stage, electrons on the uncovered anode surface are easily migrated, resulting in strong reaction kinetics and a slight increase in voltage. As discharge products gradually accumulate on the anode surface, the active reaction area becomes restricted, increasing the resistance to electron migration, which ultimately leads to a gradual decrease in voltage [50]. In summary, varying Ge content exerts a pronounced influence on the voltage stability and discharge characteristics of AZ61-1La-*x*Ge alloys across different current densities.

Figure 8c,d show the discharge performance at 2 mA cm^−2^, while Figure 8e,f correspond to the discharge performance at 10 mA cm^−2^. Under the low current density of 2 mA cm^−2^, the AZ61-1La alloy exhibits the highest anode efficiency (55.30%) and average discharge voltage (1.39 V), along with the greatest specific capacity (1130.07 mAh g^−1^) and specific energy (1994.44 mWh g^−1^). However, when correlated with the aforementioned corrosion test results, it is clear that the AZ61-1La alloy possesses the poorest corrosion resistance, characterized by significant self-corrosion behavior. Although the excessive self-corrosion of the AZ61-1La alloy may promote anodic dissolution to some extent and temporarily enhance discharge activity, such excessive self-corrosion results in inefficient consumption of the anode material [51]. These effects are detrimental to the long-term stability of battery operation, and the main factor limiting its application in actual magnesium–air batteries.

In comparison, the AZ61-1La-0.7Ge alloy demonstrates superior overall performance at the same current density. Its average discharge voltage is 1.38 V, anode efficiency is 53.43%, specific capacity is 1083.31 mAh g^−1^, and specific energy is 1908.00 mWh g^−1^. While these figures are slightly less than those of the AZ61-1La alloy, they show better stability, reflecting the advantage of a balanced trade-off between discharge performance and corrosion resistance. At a current density of 10 mA cm^−2^, the AZ61-1La-0.7Ge alloy outperforms the other three alloys, exhibiting the highest average discharge voltage (1.20 V), anode efficiency (55.58%), specific capacity (1126.83 mAh g^−1^), and specific energy (1721.38 mWh g^−1^). This outstanding performance is attributed to its sustained discharge stability and effective discharge product desorption capability.

The AZ61-1La-0.7Ge alloy exhibited excellent discharge performance under both low and high current densities. To assess its sustainability in practical applications, an intermittent discharge test was performed under a constant current density of 2 mA cm^−2^ to investigate the stability of the electrode material during repeated discharge cycles. Figure 8g illustrates the voltage variation of the AZ61-1La-0.7Ge alloy over five consecutive discharge cycles. Throughout the entire cycling process, the alloy maintained a consistently high and stable discharge voltage. The initial discharge voltage was 1.40 V. The voltage stayed constant at 1.38 V from the third cycle discharge to the fifth cycle discharge. These results indicate that the alloy maintained relatively stable discharge activity and voltage response within the limited number of test cycles. However, in future work, longer-term cycling tests are still needed to further verify the stability of its performance.

## 4. Discussion

### 4.1. Effect of Microstructure on the Properties of AZ61-1La-xGe Alloys

The microstructural features of AZ61-1La-*x*Ge alloys play a crucial role in determining their corrosion resistance and discharge behavior [52]. As can be seen from Figure 2 and Figure 3, the second phases in the alloy are mainly composed of Al_11_La3 and Mg_2_Ge. Metallographic observations indicate that the average grain size does not vary monotonically with Ge addition, but instead shows an initial reduction followed by a subsequent increase. At lower Ge contents, the alloy exhibits significant grain refinement, whereas excessive Ge (0.9 wt.%) leads to grain growth and the formation of coarse second phases. The combined action of grain refinement and the homogeneous distribution of secondary phases helps restrain localized corrosion, improves the alloy’s corrosion resistance, and reduces the tendency for chunk-effect-related material loss during anodic discharge [53,54,55]. In addition, grain refinement increases the grain boundary area, accelerating corrosion propagation along the boundaries and facilitating the formation of a relatively loose corrosion product layer. Although this may slightly reduce the overall corrosion resistance of the alloy, it benefits the detachment of corrosion products, thus maintaining the activity of the anode surface and improving discharge efficiency [56,57]. So, while the AZ61-1La-0.7Ge alloy shows slightly inferior corrosion resistance compared with the AZ61-1La-0.25Ge alloy, its fine grain structure and uniformly distributed second phases contribute to a better discharge performance. This effectively compensates for the insufficient corrosion resistance of the AZ61-1La alloy under high discharge conditions, achieving a favorable balance between corrosion resistance and discharge performance.

### 4.2. Effect of Discharge Products on the Discharge Performance of AZ61-1La-xGe Alloys

**Figure 9:**
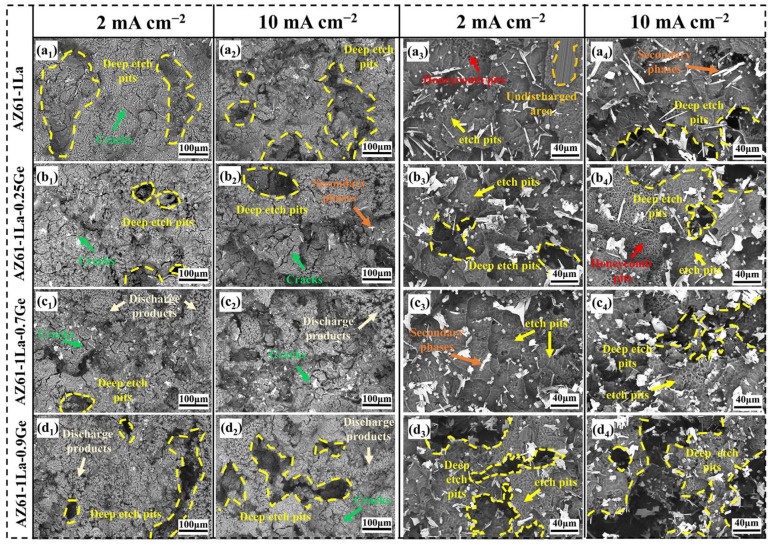
Surface morphology of AZ61-1La-*x*Ge (*x* = 0, 0.25, 0.7, 0.9 wt.%) alloys after electrical discharge, (**a_1_**–**d_1_**,**a_2_**–**d_2_**) the morphology before removal of corrosion products, while (**a_3_**–**d_3_**,**a_4_**–**d_4_**) the morphology after removal of corrosion products.

Figure 9 shows the SEM micrographs of AZ61-1La-*x*Ge alloys before and after the removal of discharge products, obtained after 10 h of discharge at various current densities. As shown in Figure 9(a_1_–d_1_,a_2_–d_2_), SEM images obtained before the removal of discharge products indicate the presence of corrosion pits on the surfaces of all AZ61-1La-*x*Ge alloys after discharging at 2 and 10 mA cm^−2^. Among them, the AZ61-1La alloy exhibits the largest pit area, while the AZ61-1La-0.9Ge alloy shows the deepest corrosion pits, indicating more severe localized dissolution. All four alloys exhibit surfaces coated with fluffy discharge products, predominantly consisting of Mg(OH)_2_. These products gradually affect the discharge behavior as the process continues. In addition, a higher applied current density accelerates the formation of discharge products, leading to a thicker product layer at 10 mA cm^−2^than at 2 mA cm^−2^ [26].

The morphology and accumulation state of discharge products significantly influence the discharge process. A loose product structure facilitates electrolyte penetration and promotes the spontaneous detachment of the discharge product, thereby exposing new active sites and enhancing the continuous discharge capability of the anode [58]. Meanwhile, cracks often appear on the product film during discharge. These cracks help mitigate the coverage effect of the discharge products, thus improving discharge activity [59]. It is evident that among the alloy samples, the AZ61-1La-0.7Ge alloy exhibits a relatively loose accumulation structure of discharge products under both current densities, with the discharge products showing pronounced large-area granular features and the most pronounced crack formation. This suggests that the discharge products exhibit strong detachability from the alloy surface, which aligns well with the high inductance (L) value observed in the impedance fitting results from electrochemical tests. The ease of product detachment in AZ61-1La-0.7Ge facilitates the exposure of more active discharge sites, contributing to its superior anodic performance. In contrast, the discharge products on the AZ61-1La and AZ61-1La-0.9Ge alloys are very dense, with few visible cracks, while the AZ61-1La-0.25Ge alloy shows obvious cracks. However, due to the dense product layer, the discharge products exhibit poor detachability from the alloy surface, thereby limiting the discharge activity.

Figure 9(a_3_–d_3_,a_4_–d_4_) presents the surface morphologies of AZ61-1La-*x*Ge (*x* = 0, 0.25, 0.7, 0.9 wt.%) alloys after removal of discharge products following 10 h of discharge at current densities of 2 mA cm^−2^ and 10 mA cm^−2^. Overall, the post-discharge corrosion morphology of the alloys is mainly characterized by shallow and smooth elliptical pits, indicating a relatively uniform discharge process. However, distinct honeycomb-like corrosion pits are observed in AZ61-1La and AZ61-1La-0.25Ge alloys. This type of morphology typically arises from intense hydrogen evolution during discharge, which induces localized corrosion [60]. Due to its intricate and irregular edges, the honeycomb structure facilitates the accumulation of discharge products and obstructs their removal, thereby reducing the active surface area of the anode. In addition, the uniformly distributed second-phase particles in the AZ61-1La-0.25Ge alloy result in the formation of a dense product film on the surface. The combined effects of a compact product film and the difficulty of product detachment significantly suppress electron exchange and magnesium dissolution during discharge, ultimately resulting in the poorest discharge performance. In contrast, the AZ61-1La-0.7Ge alloy exhibits more uniform and smoother corrosion morphologies under both current densities, without evident deep pits or honeycomb structures, indicating a more stable discharge process and a more homogeneous anodic reaction. Meanwhile, the AZ61-1La-0.9Ge alloy develops pronounced deep corrosion cavities under high current density conditions, suggesting the occurrence of a severe chunk effect. This non-uniform discharge behavior reduces the overall utilization efficiency of the anode material.

### 4.3. Effect of Second Phase on the Discharge Performance of AZ61-1La-xGe Alloys

**Figure 10:**
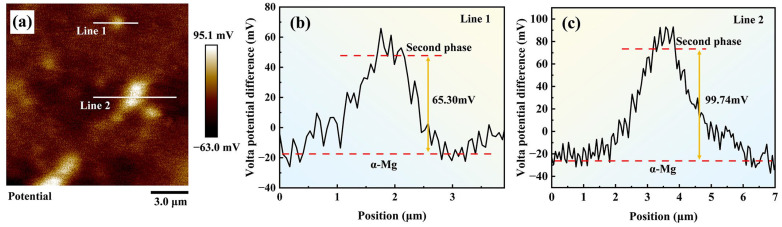
SKPFM analysis of AZ61-1La-0.7Ge alloy, (**a**) volt-potential map, (**b**,**c**) show the potential difference between α-Mg and the second phase along lines 1 and 2, respectively.

After the discharge products were removed, SEM images revealed numerous block-shaped second-phase particles dispersed across the matrix surface of the AZ61-1La-*x*Ge alloys, accompanied by distinct lamellar delamination features caused by corrosion. These features indicate that the second phase is crucial to influencing the anodic corrosion and discharge behavior. SKPFM was employed to investigate the role of the second phase in the corrosion and discharge behavior of AZ61-1La-*x*Ge alloys. Considering the similar types and morphologies of the second phases across all alloys, AZ61-1La-0.7Ge, exhibiting the best discharge performance, was selected as a representative sample for testing. The corresponding results are shown in Figure 10.

The SKPFM results reveal that the second phase exhibits a significantly higher potential than the matrix, indicating that it acts as a cathodic phase. This potential difference drives micro-galvanic corrosion between the second phase and the matrix, leading to preferential dissolution of the matrix. Variations in Ge content influence the volume fraction of the second phase, and most importantly, optimizing the Ge content is crucial to achieving a balance between anode activation and corrosion behavior. The introduction of an appropriate amount of Ge (0.7 wt.%) results in a second phase that is uniformly distributed and moderately sized within the matrix. This effectively inhibits the spread of localized corrosion while facilitating a more uniform corrosion process across the alloy, thereby improving the homogeneity of anodic discharge and effectively mitigating the occurrence of the chunk effect [61].

**Figure 11:**
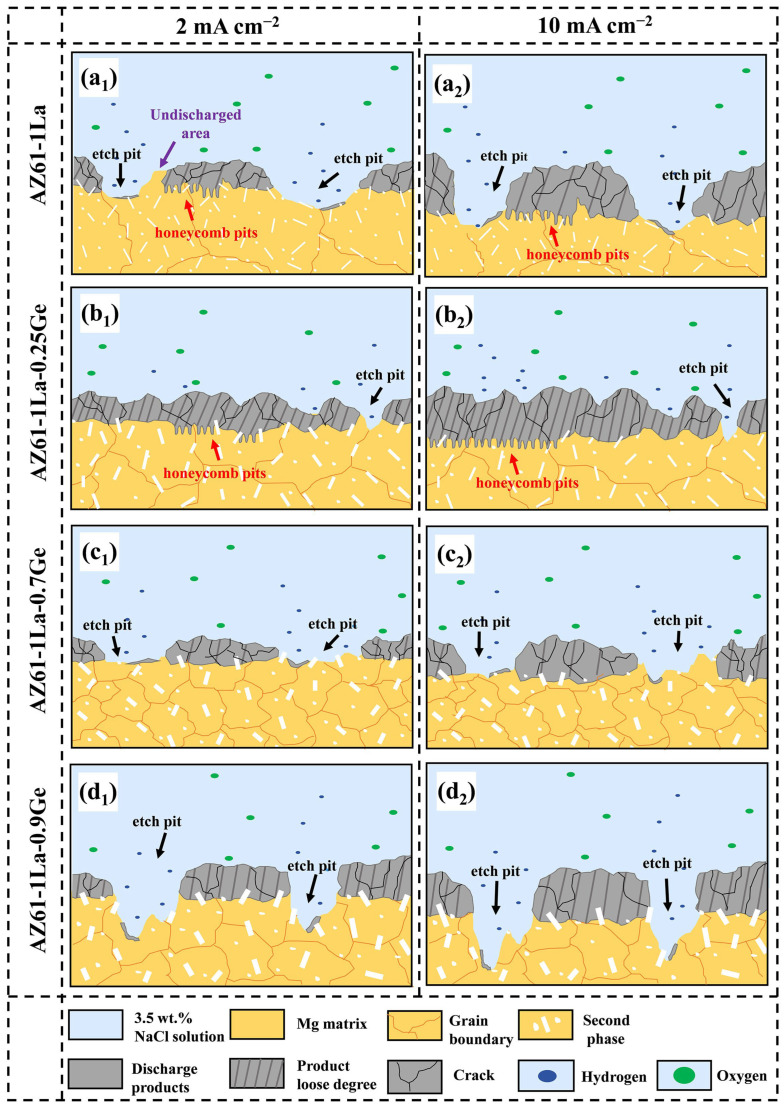
The discharge mechanism schematics of AZ61-1La-*x*Ge alloys at 2 mA cm^−2^ and 10 mA cm^−2^: (**a_1_**,**a_2_**) AZ61-1La alloy; (**b_1_**,**b_2_**) AZ61-1La-0.25Ge alloy; (**c_1_**,**c_2_**) AZ61-1La-0.7Ge alloy; (**d_1_**,**d_2_**) AZ61-1La-0.9Ge alloy.

However, when the Ge content is further increased, the second phase significantly coarsens, and its surface area fraction within the matrix rises. This leads to the formation of larger cathodic regions, disrupting the relatively balanced distribution of micro-galvanic corrosion and thereby intensifying localized corrosion [62]. Portions of the Mg matrix that have not participated in the discharge process may detach along with the second phase, resulting in non-uniform dissolution and exacerbating the chunk effect. Consequently, pronounced corrosion pits are observed on the surface of the anode, reducing the effective utilization of the anode material [63]. Furthermore, the coarsened second phase can become entangled with the discharge products, forming interwoven accumulations at the corrosion interface. This not only inhibits the self-detachment of discharge products but also impedes electrolyte penetration into the matrix [36]. Such structural features significantly compress the effective discharge area and restrict transport pathways, ultimately diminishing anodic reaction activity and leading to the poor discharge performance of the AZ61-1La-0.9Ge alloy. The discharge mechanism diagram of the AZ61-1La-*x*Ge alloy is shown in Figure 11.

Among the four alloys analyzed, the AZ61-1La-0.7Ge alloy demonstrates both outstanding discharge characteristics and superior corrosion resistance, which are primarily due to two main factors. On one hand, the introduction of an appropriate amount of Ge regulates the precipitation behavior of the second phase in the alloy, it is uniformly distributed in the matrix. This microstructural feature not only suppresses the self-corrosion tendency of the anode but also contributes to maintaining a stable discharge voltage [64]. In addition, when the second phase possesses a potential higher than that of the α-Mg matrix, a localized micro-galvanic couple can be established, which promotes preferential dissolution of the matrix and consequently increases the effective utilization of the anode material. On the other hand, Ge addition markedly refines the alloy’s grain structure, resulting in the smallest average grain size among the four alloys. Refining the grains helps the anode dissolve more evenly under discharge conditions and leads to the formation of more grain boundaries. This facilitates the formation of a looser corrosion product layer, which in turn promotes product detachment and electrolyte penetration, thereby further improving the anodic surface activity and discharge efficiency. Therefore, driven by the synergistic effect of grain refinement and second phases distribution, the AZ61-1La-0.7Ge alloy exhibits more balanced and superior overall performance. This result further confirms the critical role of Ge content in regulating second phase and grain structure, and demonstrates that the corrosion resistance and discharge performance of AZ61-1La-*x*Ge alloys can be synergistically optimized through rational design of Ge content.

## 5. Conclusions

In this study, La and varying contents of Ge (*x* = 0, 0.25, 0.7, and 0.9 wt.%) were introduced into AZ61 magnesium alloy, followed by solution treatment, to systematically investigate the microstructure, corrosion behavior, and discharge performance of AZ61-1La-*x*Ge alloys. The main conclusions are as follows:

(1) Ge content played a significant role in regulating the anode performance of AZ61-1La Mg alloys. The addition of Ge promoted the formation of the Mg_2_Ge phase and refined the grain structure, leading to an overall reduction in the corrosion rate. Although the AZ61-1La-0.25Ge alloy exhibited the best corrosion resistance, the AZ61-1La-0.7Ge alloy showed superior overall discharge performance while maintaining corrosion resistance comparable to that of the 0.25Ge alloy, indicating that an appropriate Ge addition is more beneficial for achieving a balance between corrosion resistance and discharge activity of the anode.
(2) The distribution of secondary phases and grain refinement are key factors affecting anode activation and discharge behavior. The uniform distribution of secondary phases and grain refinement jointly promote homogeneous dissolution of the alloy during discharge and effectively suppress the chunk effect. Meanwhile, grain refinement also facilitates the formation of a looser discharge product layer, thereby enhancing electrolyte penetration and improving anode utilization. The excellent discharge performance of the AZ61-1La-0.7Ge alloy is mainly attributed to its more uniform secondary-phase distribution and pronounced grain refinement.

Overall, the appropriate addition of Ge improved the coordinated optimization of corrosion resistance and discharge performance of AZ61-1La alloy anodes by promoting a more uniform distribution of secondary phases and grain refinement, thereby enhancing dissolution uniformity during discharge and improving the characteristics of the discharge product layer.

## Figures and Tables

**Table 1:** The chemical composition of the studied Mg alloy.

Alloys	Al (wt.%)	Zn (wt.%)	La (wt.%)	Ge (wt.%)	Fe (wt.%)	Cu (wt.%)	Ni (wt.%)	Mg (wt.%)
AZ61-1La	6.1990	1.3050	0.9739	-	0.0005	0.0013	0.0006	Bal.
AZ61-1La-0.25Ge	5.8290	1.2530	0.9483	0.2400	-	0.0003	0.0009	Bal.
AZ61-1La-0.7Ge	5.9740	1.2904	0.9885	0.6800	-	0.0003	0.0007	Bal.
AZ61-1La-0.9Ge	5.7740	1.2030	1.0257	0.9200	-	0.0014	0.0008	Bal.

**Table 2:** Element composition of the marked area in the EDS mappings.

Point	Mg (at.%)	Al (at.%)	Zn (at.%)	La (at.%)	Ge (at.%)	Possible Phase
A	7.80	80.75	0.18	11.27	-	Al-La
B	11.98	63.83	4.83	19.36	-	Al-La
C	81.77	3.84	0.58	0.11	13.70	Mg-Ge
D	10.87	79.60	-	9.33	0.20	Al-La
E	53.84	13.61	1.26	4.29	27.00	Mg-Ge
F	4.91	82.13	-	12.68	0.28	Al-La
G	64.81	1.80	0.74	-	32.65	Mg-Ge
H	34.79	45.32	2.53	16.60	0.76	Al-La

**Table 3:** Electrochemical parameters obtained from the polarization curves.

Alloys	*E*_corr_ (V)	*I*_corr_ (μA cm^−2^)	*P*_i_ (mm Year^−1^)
AZ61-1La	−1.51	47.57	1.09
AZ61-1La-0.25Ge	−1.46	14.06	0.32
AZ61-1La-0.7Ge	−1.46	15.34	0.35
AZ61-1La-0.9Ge	−1.47	24.54	0.56

**Table 4:** Fitting results of the EIS spectra.

Alloys	R_S_ (Ω cm^2^)	CPE_dl_ (10^−6^ s^n^ Ω^−1^ cm ^−2^)	n_dl_	R_ct_ (Ω cm^2^)	L (H)	R_L_ (Ω cm^2^)	R_total_ (Ω cm^2^)
AZ61-1La	11.06	8.70	0.9445	301.5	53.51	422.0	734.56
AZ61-1La-0.25Ge	8.68	9.95	0.9238	618.2	93.86	708.6	1335.48
AZ61-1La-0.7Ge	9.26	8.19	0.9507	178.1	130.5	952.0	1139.36
AZ61-1La-0.9Ge	11.49	7.09	0.9582	238.6	92.53	800.7	1050.79

## Data Availability

The original contributions presented in this study are included in the article. Further inquiries can be directed to the corresponding author.
